# The efficacy of artificial intelligence - powered scaffolding in individual acquisition efficiency of EFL in tertiary educational context

**DOI:** 10.3389/fpsyg.2025.1613285

**Published:** 2026-01-12

**Authors:** Yonggang Sun, Yinfang Wu

**Affiliations:** 1 Nanjing University of Chinese Medicine Hanlin College, Taizhou, China; 2 Guangxi Normal University College of Foreign studies, Guilin, China

**Keywords:** educational efficacy, AI-powered scaffolding, acquisition efficiency of EFL (English as a foreign language), tertiary education, quantitative data analysis

## Abstract

**Introduction:**

This study investigates the cognitive mechanisms and educational efficacy of AI-powered scaffolding in the acquisition of English as a Foreign Language (EFL) in tertiary education.Integrating the Technology Acceptance Model (TAM) and Cognitive Load Theory (CLT), the cross-disciplinary framework explores multidimensional pathways affecting the acquisition efficiency of EFL, focusing on learning efficiency of individual acquisition of EFL (LEF) of university students at all levels, and highlighting the mediating role of Cognitive Processing Depth (CPD) and moderating effects of Cognitive Ability (COA).

**Methods:**

Quantitative data analysis from university students using AI-assisted conversational tools of AI -powered scaffolding were specifically conducted via structural equation modeling (SEM) and necessary condition analysis (NCA).

**Results:**

Results indicate perceived usefulness (PU) and ease of use (PEoU) directly predict LEF, while interaction frequency of AI-assisted conversation (AIC) exerts indirect effects through CPD. Cognitive ability strengthens the relationship between AI-conversational tool usage and CPD, supporting Self-Regulated Learning theory. NCA identifies critical thresholds of AIC and PeoU for achieving effective learning outcomes, offering actionable insights for real-time educational interventions.

**Discussion:**

The findings emphasize the necessity of cognitive adaptation strategies, platform diversification, and learner-centric AI-conversational tool design. While limited by sample homogeneity and cross-sectional data, this study underscores the value of longitudinal approaches and broader socio-cognitive investigations in future research. Collectively, Such findings based on empirical evidence, advance the optimizing of AI-enhanced, cognitively attuned language learning systems.

## 1 Introduction

In recent years, the rapid advancement of artificial intelligence (AI) technologies, particularly breakthroughs in natural language processing (NLP), has garnered significant attention for the application of AI-assisted conversation tools in English language learning. Tools such as ChatGPT and Duolingo exemplify the potential of AIC to deliver personalized learning content, provide instant feedback, and offer flexible learning approaches (Panda et al., 2025). These tools demonstrate considerable promise in enhancing various aspects of language acquisition, including listening, speaking, reading, and writing (Fenuku, 2024). By simulating authentic conversational scenarios and leveraging speech recognition technologies, AIC assists learners in improving their practical language skills. Additionally, these tools adapt learning content based on learners’ proficiency levels, goals, and preferences, thereby increasing learning efficiency. The rapid development of AI has profoundly reshaped the landscape of language education, and AIC represents an innovative and promising approach to optimizing the language learning experience (Elaish et al., 2023). Through the integration of NLP and machine learning algorithms, AIC offers personalized, interactive, and adaptive learning environments that simulate real-world communication contexts, making them particularly valuable in the domain of English language learning.

Despite the promising potential of AIC, challenges remain in assessing their effectiveness and ensuring their broader adoption (Gašević et al., 2023; Yeh, 2025). First, there is a lack of systematic evaluation regarding the reliability of AIC in achieving learning outcomes, particularly in terms of their long-term impact on language acquisition. Additionally, the reliance on AI tools may inadvertently diminish learners’ autonomy and creativity (Le et al., 2024). Second, the integration of AIC into traditional classroom teaching remains underdeveloped, as educators often lack sufficient familiarity with these tools, limiting their ability to fully leverage AIC’s advantages (Oliveira et al., 2021; Singh et al., 2023). While AIC provides personalized services, its capacity to comprehend complex contexts and cultural nuances is limited, rendering it less effective in addressing the diverse needs of learners, especially those at advanced proficiency levels who may require greater depth and challenge. Privacy and ethical concerns also pose significant challenges (Dhirani et al., 2023; Santosh et al., 2021). Issues such as data security and the accuracy of AI-generated responses can affect user trust and acceptance. Furthermore, while AIC may initially stimulate learners’ interest, the novelty effect may wear off, leading to reduced motivation for sustained use. The absence of social interaction and emotional resonance further constrains the long-term appeal of AIC.

To address these challenges, empirical research utilizing Partial Least Squares Structural Equation Modeling (PLS-SEM) and Necessary Condition Analysis (NCA) is essential for investigating the mechanisms through which AIC enhances English learning efficiency and for exploring strategies to optimize and promote their application. Learning efficiency, defined as the ability to achieve desired learning outcomes with minimal cognitive resource and time investment (Phan and Ngu, 2021; Skulmowski and Xu, 2022), is a key metric for evaluating educational interventions. Existing literature suggests that AIC can enhance learning efficiency by fostering deeper cognitive processing and enriching learners’ perceived learning experiences (Murphy et al., 2023). However, the precise mechanisms through which AIC influences learning efficiency, as well as the moderating role of individual learner characteristics—such as cognitive ability—remain underexplored.

Grounded in Cognitive Load Theory and the Technology Acceptance Framework, this study examines the relationship between AI-assisted conversation and English learning efficiency (Baharloo and Miyan Baghi, 2024). Specifically, it investigates the mediating roles of perceived learning efficiency and cognitive processing depth, as well as the moderating role of cognitive ability (Huang, 2024). Using PLS-SEM and NCA, this study provides a comprehensive analysis of the direct and indirect pathways through which AIC impacts learning efficiency.

This research aims to contribute to the growing body of knowledge on AI in education by addressing three critical questions: (1) How does the frequency of AI-assisted conversations influence learners’ perceived and actual learning efficiency? (2) To what extent does cognitive processing depth mediate this relationship? (3) How does cognitive ability moderate the effect of AIC on learning outcomes?

The findings of this study will offer theoretical and practical insights into optimizing the use of AIC to enhance English learning experiences, providing valuable guidance for educators, technology developers, and policymakers.

## 2 Literature review

### 2.1 Cognitive load theory

Cognitive load theory (CLT), proposed by John Sweller in the 1980s, explores the limitations of the human cognitive system in learning processes and their impact on learning outcomes (Sweller, 1988). CLT classifies cognitive load into three types: intrinsic load, extraneous load, and germane load, emphasizing the interplay between the complexity of learning materials and learners’ prior knowledge. Intrinsic load is inherent to the content and is directly related to task difficulty (Klepsch and Seufert, 2020); extraneous load stems from the way information is presented and can be minimized through instructional design optimization; and germane load refers to the cognitive resources dedicated to knowledge integration and long-term memory formation (Seufert et al., 2007). As research on CLT has expanded, the theory has been increasingly applied in educational technology and online learning. Scholars have found that an optimal cognitive load enhances learning efficiency, while excessive cognitive load can hinder learning outcomes (Phan et al., 2017).

Sweller’s research suggests that while working memory capacity is limited, it can be extended through the use of schemas stored in long-term memory. Initially, CLT focused on instructional design strategies aimed at reducing unnecessary cognitive load to improve learning efficiency (Duy et al., 2025). Over time, the theory has been extended to encompass various instructional methods and learning environments, including multimedia learning, problem-solving approaches, and collaborative learning. More recently, with advancements in technology, CLT has been applied to AI-assisted learning, investigating how technological tools can optimize learning experiences, mitigate cognitive overload, and enhance learning efficiency (Duy et al., 2025; Khoa and Tran, 2024).

In the context of AI-assisted conversation tools, CLT provides a valuable theoretical lens for understanding how AI-driven interactions influence Perceived Usefulness (PU), Perceived Ease of Use (PEoU), and Perceived Learning Efficiency (Abdullah et al., 2016). Frequent AI-assisted dialogues offer real-time feedback and personalized learning experiences, reinforcing learners’ perception of the tool’s usefulness. This aligns with the concept of germane load in CLT, as an optimal cognitive load facilitates knowledge comprehension and application (Abdullah et al., 2016). Furthermore, as learners become increasingly familiar with AI-assisted conversation tools, the extraneous load associated with their usage decreases, thereby enhancing perceived ease of use (Lange et al., 2017; Sweller, 2010). Lastly, regular engagement in AI-mediated dialogues enables learners to apply acquired knowledge in real-world scenarios, promoting knowledge integration and transfer (Orru and Longo, 2019). This is consistent with CLT’s assertion that appropriately managed germane load contributes to improved learning efficiency.

### 2.2 Technology acceptance model

Technology acceptance model (TAM), proposed by Fred D. Davis in 1986, is a theoretical framework designed to explain and predict individuals’ acceptance of new technologies (Davis, 1989; Davis et al., 1989a; Davis et al., 1989b). Rooted in the Theory of Reasoned Action (TRA), TAM posits that an individual’s acceptance of a new technology is primarily determined by their cognitive perceptions and attitudes toward it (Davis et al., 1989a; Davis et al., 1989b). The model comprises two core constructs: Perceived Usefulness (PU) and Perceived Ease of Use (PEoU). PU refers to the extent to which an individual believes that using a particular technology will enhance their performance, whereas PEoU reflects the degree to which an individual perceives the technology as easy to use (Venkatesh et al., 2003). These two factors influence users’ attitudes and behavioral intentions, ultimately determining their acceptance of the technology.

### 2.3 Theoretical linkage

Integrating TAM with constructs like CPD and COA (from Cognitive Load Theory) is theoretically sound and supported by literature that extends TAM with cognitive and motivational variables. In this study, TAM is employed to analyze learners’ acceptance of AI-assisted conversation tools and their impact on learning efficiency (Sánchez-Prieto et al., 2020). AI-assisted conversation tools, through real-time interactions, personalized feedback, and contextual simulations, have been shown to significantly enhance both learning efficiency and user satisfaction (Otto et al., 2024). According to TAM, learners’ perceptions of PU and PEoU are critical determinants of their acceptance and continued use of AI tools. Specifically, AI-assisted tools facilitate more efficient language learning, thereby enhancing learners’ PU (Fedzechkina et al., 2012). Simultaneously, their user-friendly design and intuitive operation reduce the perceived difficulty of use, lowering the PEoU threshold and further promoting acceptance and adoption.

A mixed-method investigation by Eymur and Çetin (2024) explored preservice teachers’ acceptance of ChatGPT for metacognitive self-regulated learning and confirmed that perceived usefulness (PU), perceived ease of use (PEOU), enjoyment, and trust significantly influenced behavioral intention, reinforcing TAM’s applicability in AI-enhanced education. Similarly, a study on college-level second-language learners demonstrated strong predictive relationships between PU and PEOU and actual usage of AI tools, indicating TAM’s validity in language acquisition contexts (Wang et al., 2024). Meta-analytic evidence further supports TAM’s explanatory power. A synthesis of educator adoption studies by Rothberg et al. (2023) identified TAM as the dominant model explaining AI integration across K–12 and higher education settings. This aligns with case studies in higher education where TAM was effectively applied to evaluate instructor acceptance of AI-assisted grading systems (Salem et al., 2023). Beyond core constructs, TAM has been successfully integrated with cognitive and motivational dimensions. The model has also been employed in more complex frameworks such as TPACK-TAM hybrids to evaluate video generative AI adoption among K–12 teachers, highlighting TAM’s flexibility in capturing new AI modalities (Li et al., 2024). The theoretical robustness of TAM is further evidenced by its extended versions (TAM2, TAM3) and by its influence on later models like UTAUT, which maintain PU and PEOU as foundational constructs (Venkatesh et al., 2003).

Furthermore, this study introduces Cognitive Processing Depth (CPD) and Cognitive Ability (COA) as moderating variables to examine how individual differences influence the effectiveness of AI tools in improving learning efficiency (Bai, 2024; Low et al., 2025). By integrating TAM, this research not only elucidates the mechanisms through which AI technology contributes to education but also offers new perspectives and practical insights for the development of personalized learning strategies (Liu, 2023).

### 2.4 Hypothesis development

#### 2.4.1 AI-assisted conversation and perceived usefulness

In the context of educational technology, Perceived Usefulness (PU) is defined as a learner’s subjective perception of a tool’s effectiveness in enhancing learning performance (Davis, 1989). According to the Technology Acceptance Model (TAM), PU is one of the key determinants influencing technology adoption (Davis et al., 1989a; Davis et al., 1989b). Specifically, AI-assisted conversation tools can significantly enhance learners’ perception of usefulness through real-time feedback, contextually relevant dialogues, and personalized learning experiences.

Cognitive load management plays a crucial role in shaping learners’ PU when using AI-assisted conversation tools (Shively and Happonen, 2024). As interaction frequency of AI-assisted conversation (AIC) increases, these tools dynamically adjust dialogue content to align with learners’ proficiency levels, thereby reducing extraneous cognitive load (Liu et al., 2024). By simplifying complex problems and providing immediate error correction, AI systems help minimize learners’ cognitive burden, enabling them to focus more effectively on the learning task itself. This reduction in extraneous load directly enhances learners’ perception of the tool’s usefulness, as they can complete learning tasks more efficiently.

Furthermore, frequent AI-assisted interactions provide learners with increased practice opportunities and immediate feedback, which not only improve knowledge transferability but also foster greater trust and reliance on the tool. This sense of trust and dependence further reinforces learners’ perception of the tool’s value in improving their learning performance, thereby strengthening PU (Hu et al., 2017; Zou et al., 2023). Prior research has shown that learning tools that support active learning and personalized learning pathways significantly enhance the quality of the learning experience (Sweller, 2010). Therefore, the following hypothesis can be formulated:

*H1*: AI-assisted conversation positively influences Perceived Usefulness

#### 2.4.2 AI-assisted conversation and perceived ease of use

Perceived Ease of Use (PEoU) is defined as the extent to which users believe that a particular technology or tool is easy to operate (Davis et al., 1989a; Davis et al., 1989b). Within the Technology Acceptance Model (TAM), PEoU is recognized as a critical antecedent influencing technology adoption (Davis et al., 1989a; Davis et al., 1989b). As an educational technology, AI-assisted conversation tools play a pivotal role in shaping learners’ experiences, with their perceived ease of use directly impacting user engagement and continued usage intention.

Reducing extraneous cognitive load significantly enhances learners’ perception of a tool’s ease of use (Mayer, 2010). AI-assisted conversation tools, supported by frequent interactions and NLP capabilities, simplify complex learning tasks and mitigate potential barriers during use (Mayer, 2010; Skulmowski and Xu, 2022). Through repeated engagement with AI systems, learners gradually become familiar with their functionalities, interface, and operational logic. This growing familiarity reduces cognitive friction, thereby increasing the perceived ease of use. Additionally, the increased AI-learner conversational frequency lowers cognitive load by providing instant feedback and personalized learning paths (Cierniak et al., 2009). Such tailored support enables learners to process information more efficiently, alleviating cognitive stress caused by information overload or difficulties in comprehension (Cierniak et al., 2009). The immediacy and adaptability of AI tools further enhance their perceived usefulness (PU), as learners can directly observe how these tools facilitate goal achievement and improve learning efficiency.

The user-friendly interfaces and intuitive designs of AI tools further contribute to reducing the complexity associated with their use, thereby enhancing PeoU (Azzam and Beckmann, 2022). When learners perceive AI tools as easy to use, they are more likely to engage with them frequently, reinforcing their perception of the tools’ usefulness. Moreover, by minimizing cognitive barriers during the learning process, AI tools enable learners to focus more effectively on the content itself, ultimately improving their perceived learning efficiency. This enhanced perception of efficiency, in turn, fosters greater AI tool adoption, creating a positive feedback loop.

Cognitive Load Theory provides a valuable perspective for understanding how AI-assisted conversation tools enhance English learning efficiency through their influence on PeoU (Gil et al., 2021). By reducing unnecessary cognitive load, AI tools optimize the learning process, allowing learners to allocate their cognitive resources more effectively and thereby improve learning outcomes (Monostori, 2003). Therefore, the following hypotheses can be derived:

*H2*: AI-assisted conversation positively influences Perceived Ease of Use (PEoU)

#### 2.4.3 Perceived usefulness and learning efficiency

Learning efficiency (LEF) is defined as learners’ perception of their ability to achieve learning objectives within a given timeframe and with a specific level of cognitive resource investment. An increase in learners’ Perceived Usefulness (PU) of a tool is often accompanied by an enhanced sense of learning efficiency (Phan and Ngu, 2021). For instance, when learners believe that a tool facilitates faster knowledge or skill acquisition—through features such as real-time feedback, personalized learning paths, or optimized instructional design—they are more likely to perceive an improvement in their learning efficiency.

Perceived usefulness refers to learners’ subjective perception of a tool or technology’s effectiveness in enhancing learning outcomes. Within the framework of the Technology Acceptance Model (TAM), PU is considered a critical predictor of technology adoption and use (FakhrHosseini et al., 2024). When learners perceive that a tool significantly improves their learning outcomes, they are more inclined to engage with it actively (Pan and Jordan-Marsh, 2010). This engagement, in turn, can directly or indirectly influence learning efficiency.

The key mechanism through which PU affects learning efficiency lies in its role in managing cognitive load (Pu et al., 2019). Specifically, tools perceived as useful are typically designed to optimize the learning process and resource allocation, thereby reducing extraneous load and enhancing germane load (Hearrington, 2010). For example, an AI-assisted conversation tool that is deemed useful can provide automated real-time feedback and personalized learning recommendations, minimizing unnecessary cognitive distractions and allowing learners to focus on core learning content (Pu and Chang, 2023). This process of reducing extraneous interference while reinforcing relevant cognitive efforts directly contributes to improved learning efficiency.

When learners perceive that a tool directly supports their learning objectives, they tend to exhibit higher motivation and engagement (Rahman et al., 2023). This increased engagement fosters deeper cognitive processing and knowledge transfer, ultimately enhancing knowledge retention and application. Prior research has demonstrated that PU significantly influences learners’ positive attitudes toward learning activities and their willingness to exert effort, both of which are crucial for improving learning efficiency (Davis, 1989; Davis et al., 1989a; Davis et al., 1989b; Sweller, 2010; Venkatesh et al., 2003)

*H3*: Perceived Usefulness (PU) positively influences Learning Efficiency (LEF)

#### 2.4.4 Perceived ease of use and learning efficiency

Perceived ease of use (PEoU) is defined as the extent to which learners perceive a tool or technology as easy to operate, understand, and learn (Davis, 1989; Davis et al., 1989a; Davis et al., 1989b). Within the Technology Acceptance Model (TAM), PEoU not only influences learners’ attitudes toward technology but also has both direct and indirect effects on Learning Efficiency (LEF) (De Smedt et al., 2010). The relationship between PEoU and LEF lies in the ability of an intuitive and user-friendly tool to reduce learners’ cognitive burden associated with technology operation, thereby allowing more cognitive resources to be allocated to core learning tasks (De Smedt et al., 2010; Shin and Chan, 2004).

A key impact of high PEoU is the significant reduction of extraneous cognitive load (Kumar and Mohite, 2018). For instance, when learners engage with an AI-assisted conversation tool that features an intuitive interface, clear functionality, and seamless interaction, they require minimal cognitive effort to understand and navigate the system (Hu et al., 2017). By reducing the extraneous load associated with tool operation, learners can concentrate on the learning content itself, directly enhancing their learning efficiency (Skulmowski and Xu, 2022).

Moreover, a high level of PEoU fosters learners’ confidence and engagement, aligning with Cognitive Load Theory (CLT), which highlights the benefits of germane load in knowledge processing. A user-friendly tool facilitates a seamless learning experience, increasing the likelihood of learners entering a flow state—a psychological state in which cognitive resources are optimally utilized, leading to enhanced knowledge processing and integration. Additionally, studies have shown that high PEoU can alleviate frustration and anxiety, further improving learning efficiency by enabling learners to complete tasks in a more positive psychological state (Davis, 1989; Davis et al., 1989a; Davis et al., 1989b; Sweller, 2010; Venkatesh et al., 2003). Thus, the following hypothesis can be proposed:

*H4*: Perceived Ease of Use (PEoU) positively influences Learning Efficiency (LEF)

#### 2.4.5 Cognitive ability and learning efficiency

Cognitive ability (COA) refers to an individual’s capacity for information processing (Kumar and Mohite, 2018), problem-solving, and knowledge integration, encompassing key functions such as memory, attention, reasoning, and executive functioning (Moiseenok and Kanunnikova, 2023). High learning efficiency is typically characterized by achieving greater learning outcomes with reduced time and effort. Control of Attention (COA) was selected as a moderator based on its central role in self-regulated learning frameworks and in Technology-Enhanced Learning studies (Bannert, 2002). Prior research suggests that learners with higher attentional control are better able to benefit from cognitively demanding tools, such as conversational AI, especially under high task complexity (Gazzaley and Rosen, 2016). This supports the logic that COA moderates the influence of CPD on learning effectiveness (LEF). Learners with high cognitive ability exhibit superior knowledge integration and problem-solving skills, enabling them to quickly comprehend new information, assimilate it with prior knowledge, and maintain high performance in complex tasks (Sweller, 2010). This ability significantly reduces intrinsic load while enhancing germane load, thereby directly improving learning efficiency.

Furthermore, learners with higher cognitive ability tend to possess stronger metacognitive and self-regulated learning skills. They can effectively plan, monitor, and evaluate their learning processes, allowing them to optimize learning strategies and complete tasks efficiently (Sweller, 2010). This self-regulatory capacity further enhances learning efficiency by enabling learners to allocate attention and resources effectively, minimizing unnecessary cognitive effort. Research has shown that high cognitive ability allows learners to filter irrelevant information more efficiently and focus on key concepts, thereby reducing extraneous cognitive load (Sweller, 2010). Additionally, such learners are more adept at leveraging learning support systems (e.g., AI-based tools or instructor guidance) to facilitate knowledge construction and transfer. Thus, the following hypothesis can be proposed:

*H5*: Cognitive Ability (COA) positively influences Learning Efficiency (LEF)

#### 2.4.6 Perceived ease of use and perceived usefulness

Perceived Ease of Use (PEoU) refers to the extent to which users believe that a particular technology or tool is easy to understand, learn, and operate (Davis, 1989; Davis et al., 1989a; Davis et al., 1989b). Perceived Usefulness (PU), on the other hand, reflects users’ subjective perception of whether a technology or tool can effectively enhance their work, learning, or daily efficiency (Davis et al., 1989a; Davis et al., 1989b). Within the framework of the Technology Acceptance Model (TAM), PEoU serves as a key antecedent variable influencing PU.

According to the theoretical assumptions of TAM, the ease of use of a tool or technology directly affects users’ evaluation of its usefulness. Specifically, when users perceive a tool as easy to use, they are more likely to believe that it can help them complete tasks more efficiently (Cho et al., 2009). A high level of ease of use reduces users’ cognitive load when operating the tool, allowing them to focus more on their core tasks, thereby reinforcing their perception of the tool’s utility (Jegundo et al., 2020). For instance, an intuitive and user-friendly learning platform enables learners to quickly grasp its functionalities, allowing them to concentrate on the learning content itself. This enhanced focus further strengthens their perception of the platform’s usefulness.

Empirical studies have demonstrated that PEoU enhances users’ positive attitudes toward technology and facilitates its adoption by reducing psychological barriers such as learning costs and operational complexity (Davis et al., 1989a; Davis et al., 1989b). Davis et al. (1989a) and Davis et al. (1989b) posited that when users perceive a technology as easy to use, they are more likely to develop higher expectations regarding its usefulness, as ease of use makes the benefits of the technology more readily accessible (Salend, 2009). For example, if users find an AI-assisted conversation tool intuitive and require minimal training to operate, they are more inclined to perceive it as a practical tool for improving learning efficiency or problem-solving (Huq et al., 2024). Thus, the following hypothesis can be proposed:

*H6*: Perceived Ease of Use (PEoU) positively influences Perceived Usefulness (PU).

#### 2.4.7 Cognitive processing depth as a moderator between AI-assisted conversation and perceived usefulness

AI-assisted conversation (AIC) refers to the frequency with which learners interact with AI-powered conversational tools within a given period (Jiang, 2025). Frequent engagement with these tools provides immediate feedback, personalized guidance, and context-aware learning experiences, thereby enhancing learners’ Perceived Usefulness (PU)—the extent to which they perceive the tool as effective in helping them achieve their goals (Huang, 2024). Cognitive Processing Depth (CPD) represents the depth of cognitive engagement learners reach when processing information. According to Lockhart and Craik (1990) Levels of Processing Theory, deeper cognitive processing (e.g., analysis, evaluation, and synthesis) generally leads to better learning outcomes, whereas shallow processing (e.g., rote memorization or simple repetition) may yield more limited effects (Baddeley, 1978).

In a learning environment, CPD may moderate the impact of AIC on PU (Bergeron et al., 2022). The depth of learners’ cognitive engagement determines the extent to which they benefit from frequent AI-assisted conversations (Yazdi et al., 2017). When learners engage in deeper cognitive processing, frequent AI interactions can facilitate a more profound understanding of complex concepts and problem-solving (Ouyang et al., 2023), thereby significantly enhancing their perception of the tool’s usefulness. Conversely (Joksimovic et al., 2023), if learners engage only in shallow processing, they may fail to fully leverage the advantages of AI-assisted interactions, leading to a more limited increase in their perceived usefulness of the tool.

According to the Technology Acceptance Model (TAM), the frequency of tool usage is positively associated with users’ perceived usefulness of the tool (Davis, 1989; Davis et al., 1989a; Davis et al., 1989b). Frequent AI-assisted conversations provide immediate feedback and dynamically adjust learning tasks, enabling users to achieve their learning objectives more efficiently, thereby strengthening PU (Izadi and Forouzanfar, 2024). However, the strength of this positive relationship may depend on the depth of learners’ cognitive processing (Jegede, 2024; Pari, 2024).

When learners engage in deep cognitive processing (e.g., analysis, reasoning, and knowledge integration), frequent AI interactions offer critical support by fostering deeper comprehension of learning content. In such cases, AI’s personalized feedback and complex problem-solving capabilities become particularly valuable, substantially enhancing PU (Muthmainnah et al., 2022). In contrast, if learners engage only in shallow cognitive processing (e.g., simple recall or mechanical exercises), they may not fully utilize the tool’s interactive features (Sánchez et al., 2005). As a result, even with frequent AI-assisted conversations, the perceived usefulness of the tool may improve only marginally, as learners struggle to translate AI-generated insights into meaningful learning outcomes.

According to Cognitive Load Theory (CLT) proposed by Sweller (1988, 2010), deep cognitive processing effectively manages and utilizes germane load—the cognitive resources dedicated to meaningful learning and schema construction. Under high-CPD conditions, frequent AI-assisted interactions enhance learners’ germane load, leading to a more significant improvement in their perceived usefulness of the tool (Chen and Chang, 2024; Schulz et al., 2024). Conversely, in low-CPD conditions, learners may focus primarily on surface-level information, resulting in a weaker enhancement of PU (Feng, 2025). Thus, the following hypothesis can be proposed:

*H7*: Cognitive Processing Depth (CPD) moderates the relationship between AI-assisted conversation and Perceived Usefulness (PU).

#### 2.4.8 Cognitive processing depth moderate AI-assisted conversation and perceived ease of use

Based on the theoretical foundations of the Technology Acceptance Model (TAM), user interaction frequency with a technology is expected to have a positive impact on their perception of its Perceived Ease of Use (Davis, 1989). In the context of AI-assisted conversations, frequent interactions are likely to increase users’ familiarity with the tool’s interface, features, and functionality, reducing the cognitive load associated with its use. This increased familiarity should lead to a more favorable perception of the tool’s ease of use. The direct link between frequent AI - assisted conversations and PEoU, frequent user - technology interaction often boosts PEoU (Davis, 1989). In AI - assisted dialogue settings, frequent interactions can increase users’ familiarity with the tool’s interface, features, and functions, reducing usage - related cognitive load (Quiroga et al., 2004). This heightened familiarity should lead to a more favorable PEoU.

Deeper cognitive processing usually improves learning outcomes, while shallow processing often reduces learning efficiency. When learners process information deeply, frequent AI - assisted conversations can be seen as an effective learning - goal - supporting tool. In this case, frequent AI interactions, with timely feedback, personalized support, and contextualized learning experiences, can facilitate complex problem - solving and understanding, thereby enhancing PeoU (Mayer and Moreno, 2003). When learners process information shallowly, frequent AI interactions may be seen as tedious or superfluous, increasing perceived operational burden and lowering PEoU. Here, the repetitiveness of interactions does not significantly contribute to deeper learning, resulting in a less favorable evaluation of the tool’s usability. Thus, the following hypothesis can be proposed:

*H8*: Cognitive Processing Depth (CPD) moderates the relationship between AI-assisted conversation and Perceived Ease of Use (PEoU).

#### 2.4.9 Cognitive ability moderates the relationship between AI-assisted conversation and perceived ease of use

According to the Technology Acceptance Model (TAM), frequent interactions between users and a tool can enhance familiarity and reduce perceived difficulty, thereby strengthening the perception of the tool’s ease of use. However, individual differences in cognitive ability may lead to variations in the strength of this relationship (Carroll, 1978). Users with higher cognitive ability are able to quickly learn and adapt to the tool’s features and operational logic. Even with frequent interactions, they can efficiently manage the complexity of the tool and derive a more positive experience (Pellegrino and Glaser, 1979). As a result, frequent AI-assisted conversations are more likely to significantly enhance their perception of the tool’s ease of use.

On the other hand, users with lower cognitive ability may face greater cognitive load during frequent interactions, particularly when the tool’s operation is complex or requires substantial learning (Boogert et al., 2018). These users are more likely to feel confused or fatigued, which can reduce their perception of the tool’s ease of use. According to cognitive psychology theory, cognitive ability influences the time required for users to learn and adapt to new technologies. Users with higher cognitive ability demonstrate stronger adaptability and learning capacity when faced with complex tasks. Therefore, they can more rapidly achieve familiarity and reduce perceived difficulty through frequent interactions. In contrast, users with lower cognitive ability may require more time to adapt, diminishing the positive impact of frequent interactions on their perception of ease of use. Thus, the following hypothesis can be proposed:

*H9*: Cognitive Ability (COA) moderates the relationship between AI-assisted conversation and Perceived Ease of Use (PEoU).

**Figure 1:**
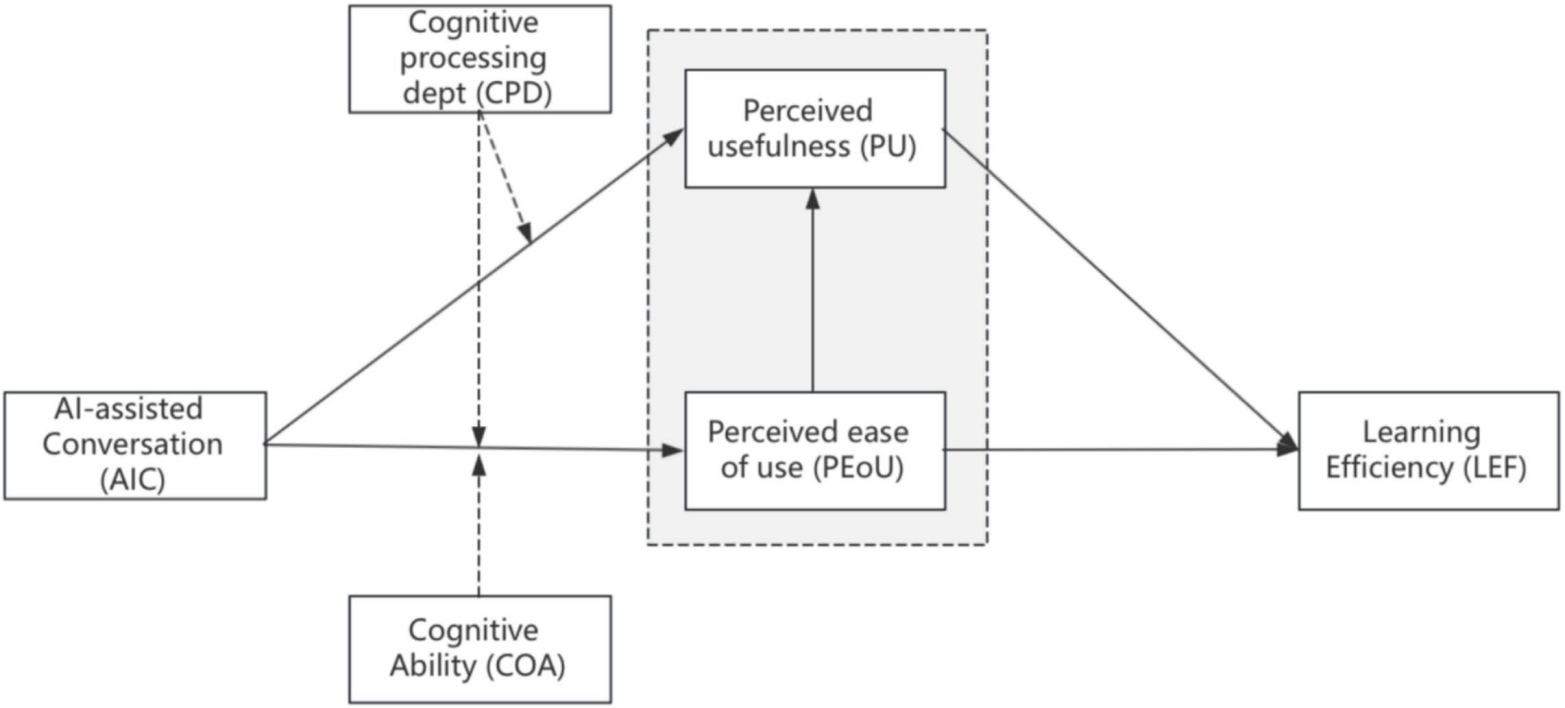
Conceptual framework.

Based on the above hypotheses, Therefore, the conceptual model of this study is as figure 1,adapted by Cognitive Load Theory (Sweller, 1988). (Figure 1).

## 3 Methodology

### 3.1 Research design

This study will employ Partial Least Squares Structural Equation Modeling (PLS-SEM) to analyze the relationships between constructs within the research model (Ringle et al., 2020). PLS-SEM is chosen because it can handle complex models with multiple constructs and is well-suited for exploratory research. The study will assess the reliability and validity of the measurement model, including reliability indicators (Cronbach’s alpha, reported for reference, and composite reliability as a more robust index of internal consistency), convergent validity (using the Average Variance Extracted, AVE), and discriminant validity (using the Fornell-Larcker criterion and the Heterotrait-Monotrait ratio, HTMT). The structural model will be evaluated to test the hypothesized relationships between constructs. Path coefficients, t-values, and R^2^ values will be examined to determine the strength and significance of these relationships. Bootstrapping with 5,000 subsamples will be used to assess whether the estimated structural paths are statistically significant (Streukens and Leroi-Werelds, 2016). Additionally, Necessary Condition Analysis (NCA) will be conducted to identify the critical conditions under which AI-assisted tools enhance learning efficiency. This dual-method approach strengthens the robustness of the findings by addressing both statistical association and necessary conditions.

To collect data, a structured questionnaire was designed using validated scales adapted from existing literature. The questionnaire consists of four sections: (1) demographic information, (2) frequency and context of AI-assisted tool usage, (3) perceived usefulness and ease of use of the tool, and (4) self-reported learning efficiency, cognitive processing depth, and cognitive ability. All items are measured on a five-point Likert scale, ranging from “strongly disagree” to “strongly agree.” The target population includes English learners with experience using AI-assisted conversational tools. A purposive sampling technique is employed to ensure that participants have relevant experience with the tools under investigation.

### 3.2 Data collection

In this study, data collection was conducted via an online questionnaire hosted by Wenjuanxing, a widely recognized survey platform in China. Wenjuanxing provides access to a diverse participant pool exceeding 2.6 million respondents, offering specialized paid sampling services that enhance both sample representativeness and data reliability (Wang et al., 2021). To ensure that survey respondents accurately matched the targeted user characteristics, the platform incorporated screening procedures at the survey outset. These screening questions effectively filtered potential respondents, guaranteeing that participants included in the final sample had relevant experience with AI-assisted conversational tools in English language learning contexts.

To further strengthen data quality and validity, multiple quality-control strategies were employed (Su et al., 2024). Firstly, the platform utilized automated mechanisms to detect and remove potentially invalid responses, such as those from duplicate IP addresses, participants completing the survey in unusually short times, or respondents exhibiting suspicious answering behaviors (Lu et al., 2021). Secondly, only responses completed within a realistic and reasonable duration were retained for subsequent analysis; this step ensured that the data represented thoughtful and authentic participant engagement (Wang et al., 2021). Thirdly, attention-check questions were strategically embedded throughout the survey to identify and exclude inattentive or careless respondents.

Moreover, ethical standards were strictly upheld throughout the research process, including obtaining informed consent from participants, emphasizing voluntary participation, and ensuring anonymity and confidentiality during data collection and analysis. The rigorous sampling approach and comprehensive data validation techniques provided by the Wenjuanxing platform contributed substantially to the overall reliability and representativeness of the dataset, thereby laying a solid methodological foundation for subsequent empirical analyses.

## 4 Results

### 4.1 Demographic characteristics

The target population of this study consisted exclusively of undergraduate and graduate students enrolled at University. We distributed the online questionnaire via the platform and student mailing lists, ensuring voluntary participation. A total of 330 questionnaires were collected, of which 297 were valid after data cleaning. Thus, the analyses were conducted based on these 297 responses. Table 1 presents the demographic characteristics of the participants, including gender, age, and frequency of AI-assisted tool usage.

**Table 1:** Demographic characteristics.

Category	Gender	Age	Use frequency
A. Male	90(30.3%)		
B. Female	207(69.7%)		
A.18–20		99(33.3%)	
B.21–22		38(12.8%)	
C.23–25		21(7.1%)	
D.26 or above		139(46.8%)	
A. 0 never			34(11.4%)
B. 1–3.			85(28.6%)
C. 4–8.			111(37.4%)
D. 9–15.			50(16.8%)
E. 16 or above			17(5.7%)

*N* = 297; Values in the parentheses are proportions.

Regarding gender, 207 respondents (69.7%) were female, while 90 respondents (30.3%) were male, indicating a notable gender imbalance. In terms of age distribution, 46.8% of participants were 26 years or above, followed by 33.3% aged 18–20, 12.8% aged 21–22, and 7.1% aged 23–25, suggesting that the majority of the sample consisted of students in Tertiary Education encompassing all levels.

In terms of AI-assisted tool usage, the largest proportion of participants reported using such tools 4–8 times per week (37.4%, *n* = 111), followed by 1–3 times per week (28.6%, *n* = 85), 9–15 times per week (16.8%, *n* = 50), and 16 times or more (5.7%, *n* = 17). Notably, 11.4% (*n* = 34) indicated that they had never used AI-assisted tools.

### 4.2 PLS-SEM result

Empirical analysis is typically conducted using PLS-SEM. PLS-SEM does not impose distributional requirements on data and can accommodate extended models based on existing theories (Hair et al., 2021). PLS-SEM combines factor analysis and multiple regression modeling to explore the path relationships between observed and unobserved variables. Therefore, this study employs PLS-SEM for empirical analysis and utilizes Smart-PLS 4.0 software. Smart-PLS offers high estimation accuracy and user-friendly data processing operations.

### 4.2.1 Assessing the outer measurement model

#### 4.2.1.1 Reliability and convergent validity

The strength and quality of a measurement model are typically determined by assessing its reliability and validity. Reliability analysis focuses primarily on examining the internal consistency and stability of the questionnaire items. Commonly reported reliability indicators include Cronbach’s alpha and composite reliability, although Cronbach’s alpha should be interpreted cautiously and not as a direct measure of internal consistence (Sarstedt et al., 2014b). The internal consistency of the measurement instrument was carefully examined. The results presented in Table 2 that all Cronbach’s alpha values calculated from the data exceed the recommended threshold of 0.7. Similarly, the composite reliability scores for each construct are also higher than the accepted standard of 0.7. These outcomes collectively confirm that the questionnaire used in this research exhibits robust internal consistency and reliable measurement properties, thereby supporting the subsequent validity analyses and structural model evaluation.

**Table 2:** Composite reliability and convergent validity of the measurement model.

Construct	Item	Loading	VIF	rho_A	CR	AVE
AIC	AIC1	0.791	1.803	0.853	0.895	0.629
	AIC2	0.765	1.699			
	AIC3	0.809	1.917			
	AIC4	0.797	1.795			
	AIC5	0.804	1.909			
COA	COA1	0.805	1.92	0.861	0.899	0.641
	COA2	0.812	1.949			
	COA3	0.824	2.07			
	COA4	0.774	1.751			
	COA5	0.788	1.792			
CPD	CPD1	0.788	1.714	0.847	0.888	0.614
	CPD2	0.753	1.692			
	CPD3	0.841	2.597			
	CPD4	0.723	1.594			
	CPD5	0.806	2.308			
LEF	LEF1	0.818	1.962	0.873	0.906	0.659
	LEF2	0.833	2.202			
	LEF3	0.799	1.812			
	LEF4	0.784	1.911			
	LEF5	0.822	2.049			
PEoU	PEoU1	0.819	2.068	0.872	0.907	0.661
	PEoU2	0.787	1.821			
	PEoU3	0.814	2.131			
	PEoU4	0.794	1.814			
	PEoU5	0.849	2.463			
PU	PU1	0.851	2.023	0.879	0.914	0.727
	PU2	0.824	1.848			
	PU3	0.879	2.648			
	PU4	0.855	2.497			

To ensure the reliability and convergent validity of the constructs, we evaluated the measurement model using several key statistical indicators, including factor loadings, Variance Inflation Factors (VIF), Cronbach’s *α*, rho_A, Composite Reliability (CR), and Average Variance Extracted (AVE). The results are summarized in Table 2 and demonstrate that the measurement items used in this study exhibit strong reliability and sufficient convergent validity.

Factor loadings for all items ranged from 0.723 to 0.879, exceeding the commonly accepted threshold of 0.70 (Hair et al., 2019), indicating that each item adequately reflects its corresponding construct. The VIF values varied between 1.594 and 2.648, which are well below the critical threshold of 5.0 (Kock, 2015), suggesting that there is no significant issue of multicollinearity among the observed variables. We reported Cronbach’s alpha for each construct, but relied primarily on composite reliability to evaluate internal consistency, as it better accounts for factor loadings and measurement error. Cronbach’s α values ranged from 0.842 to 0.875, while CR values ranged from 0.888 to 0.914. Both sets of results are above the recommended threshold of 0.70 (Considine et al., 2005), indicating that the constructs demonstrate excellent internal consistency.

In addition to Cronbach’s alpha and composite reliability (CR), we report (rho_A) by Dijkstra and Henseler (2015), which is recommended for assessing internal consistency reliability in PLS-SEM. Unlike Cronbach’s alpha, which assumes equal loadings among indicators, rho_A considers the actual construct loadings and thus provides a more precise reliability estimate. A rho_A value above 0.70 indicates acceptable reliability, while values exceeding 0.80 reflect high construct consistency (Dijkstra and Henseler, 2015).

#### 4.2.1.2 Fornell-Larcker criterion and the Heterotrait-Monotrait (HTMT) ratio test

Convergent validity was assessed based on the Average Variance Extracted (AVE). All constructs achieved AVE values between 0.614 and 0.727, which are above the recommended minimum threshold of 0.50 (Fornell and Larcker, 1981). This result suggests that more than 50% of the variance in the observed variables is explained by the underlying latent construct, demonstrating satisfactory convergent validity (Fornell and Larcker, 1981). Thus confirming strong convergent validity. Moreover, discriminant validity was evaluated using both the Fornell-Larcker criterion and the Heterotrait-Monotrait (HTMT) ratio test, with results generated through PLS-SEM software analysis. According to the Fornell-Larcker criterion, discriminant validity is present when the square root of the AVE for each construct is larger than its correlation with other constructs. As demonstrated by the results summarized in Tables 3, 4, this condition was satisfied. Additionally, discriminant validity was further supported by the HTMT ratio analysis, with all HTMT values among the studied constructs falling below the established threshold of 0.85 (Henseler et al., 2015). Collectively, these findings confirm that the measurement model possesses adequate discriminant validity, ensuring that the constructs measured by the questionnaire are statistically distinct.

**Table 3:** Discriminant validity assessment using HTMT criterion.

Construct	AIC	COA	CPD	LEF	PEoU	PU	CPDx AIC	COAx AIC
AIC								
COA	**0.624**							
CPD	0.684	**0.568**						
LEF	0.756	0.68	**0.713**					
PEoU	0.625	0.591	0.568	**0.708**				
PU	0.279	0.25	0.242	0.333	**0.303**			
CPD x AIC	0.313	0.211	0.284	0.275	0.165	**0.047**		
COA x AIC	0.343	0.097	0.217	0.186	0.183	0.129	**0.635**	

The off-diagonal values in the above matrix are the square correlations between the latent constructs and the diagonals (bold) are AVEs.

**Table 4:** Discriminant validity assessment using Fornell–Larcker criterion.

	AIC	COA	CPD	LEF	PEoU	PU
AIC	**0.793**					
COA	0.535	**0.801**				
CPD	0.58	0.484	**0.783**			
LEF	0.65	0.587	0.611	**0.812**		
PEoU	0.54	0.514	0.488	0.62	**0.813**	
PU	0.243	0.221	0.211	0.295	0.268	**0.853**

The off-diagonal values in the above matrix are the square correlations between the latent constructs and the diagonals (bold) are AVEs.

#### 4.2.1.3 Correlation matrix of key study variables

To further examine the linear associations among the core study constructs, a Pearson correlation analysis was conducted, and the results are presented in Table 5. As expected, all variables showed positive and statistically significant associations, indicating robust interrelationships among the constructs. Specifically, AI-assisted interaction count (AIC) exhibited the strongest correlations with learning effectiveness (LEF, *r* = 0.650), cognitive processing depth (CPD, *r* = 0.580), and cognitive awareness (COA, *r* = 0.535), suggesting that higher levels of AI interaction are associated with enhanced cognitive and learning outcomes. LEF also demonstrated strong associations with both COA (*r* = 0.587) and CPD (*r* = 0.611), supporting the theoretical expectation that deeper cognitive engagement enhances learning experiences. In addition, perceived ease of use (PEoU) was moderately correlated with LEF (*r* = 0.620) and AIC (*r* = 0.540), implying that participants who found AI tools easier to use also reported greater interaction and improved learning outcomes. Although perceived usefulness (PU) showed comparatively lower correlations, it still exhibited meaningful positive relationships with AIC (*r* = 0.243), COA (*r* = 0.221), and LEF (*r* = 0.295), suggesting its potential indirect influence on learning outcomes.

**Table 5:** Correlation matrix of key study variables (*n* = 297).

	M	SD	1	2	3	4	5	6
AIC	4.21	0.56	1	–	_	_	_	_
COA	4.05	0.62	.535	1	_	_	_	_
CPD	4.11	0.58	.580	.484	1	_	_	_
LEF	3.97	0.66	.650	.587	.611	1	_	_
PEoU	3.88	0.69	.540	.514	.488	.620	1	_
PU	4.03	0.61	.243	.221	.211	.295	.268	1

*n* = 297; critical value of Pearson’s *r* = 0.11 (*p* < 0.05, two-tailed). All correlations above this threshold are statistically significant. No multicollinearity concerns were detected (all *r* < 0.80).

These results provide initial empirical justification for subsequent path modeling. No multicollinearity concerns were observed, as all correlation coefficients remained well below the critical threshold of 0.80 (Kline, 2015), ensuring statistical independence among latent variables in the structural equation modeling phase.

#### 4.2.2 Structural model testing

Following this verification, a bootstrapping procedure involving 5,000 resamples was conducted to determine whether the estimated path coefficients are statistically significant and robust using bootstrapping (Hair et al., 2021).

Based on the results in Table 6, the structural model demonstrates several significant relationships. Algorithmic information control (AIC), cognitive awareness (COA), and cognitive dependence (CPD) all have positive effects on perceived ease of use (PEoU), with significant paths (AIC → PEoU, *T* = 4.518, *p* < 0.001; COA → PEoU, *T* = 4.596, *p* < 0.001; CPD → PEoU, *T* = 3.414, *p* < 0.001). PEoU significantly influences perceived usefulness (PU) (PEoU → PU, *T* = 2.447, *p* < 0.05) and learning effectiveness (LEF) (PEoU → LEF, *T* = 14.308, *p* < 0.001), while PU also positively impacts LEF (PU → LEF, *T* = 2.738, *p* < 0.01).

**Table 6:** Assessment of structural model.

	Coefficient	Std. error	T statistics	*p-*values
AIC - > PEoU	0.277	0.28	4.518	0.00
COA - > PEoU	0.278	0.281	4.596	0.00
CPD - > PEoU	0.199	0.196	3.414	0.00
PEoU, *R*^2^ = 0.389; *Q*^2^ predict = 0.249				
AIC - > PU	0.12	0.125	1.571	0.03
CPD - > PU	0.063	0.066	0.816	0.415
PEoU - > PU	0.177	0.176	2.447	0.045
PU, *R*^2^ = 0.088; *Q*^2^ predict = 0.058				
PU - > LEF	0.139	0.141	2.738	0.057
PEoU - > LEF	0.583	0.584	14.308	0.061
LEF, *R*^2^ = 0.402; *Q*^2^ predict = 0.258				
CPD x AIC - > PEoU	0.06	0.059	1.147	0.252
CPD x AIC - > PU	0.026	0.027	0.456	0.649
COA x AIC - > PEoU	−0.054	−0.053	0.973	0.331

However, AIC → PU and CPD → PU are not significant, suggesting that their influence on PU operates indirectly through PEoU. The model explains moderate variance in PEoU (*R*^2^ = 0.389), but PU’s variance is relatively low (*R*^2^ = 0.088). Finally, moderation effects (CPD × AIC, COA × AIC) are insignificant, indicating no interactive impact on PEoU or PU. Overall, the findings highlight the central role of PEoU as a mediator driving both PU and LEF, while suggesting that future research should explore additional factors to strengthen the explanatory power for PU.

### 4.3 Necessary condition analysis

Necessary condition analysis (NCA) is an innovative research method explicitly designed to identify necessary conditions within complex statistical associations—that is, conditions that are indispensable for the occurrence of an outcome variable (Dul, 2016b). Unlike traditional analytical approaches such as regression or structural equation modeling, NCA not only identifies the existence of necessary conditions but also quantifies their strength and scope of constraint, thereby effectively uncovering the distinctive “necessary but not sufficient” relationships between independent and dependent variables. By calculating the minimal levels of necessary conditions required to achieve specific outcome thresholds—known as “ceiling lines”—NCA precisely pinpoints bottleneck factors and assesses their effect sizes (Radanliev, 2025; Ye and Kuang, 2025). Thus, it effectively complements traditional sufficiency analyses and provides a more comprehensive explanatory framework for causal mechanisms.

**Figure 2:**
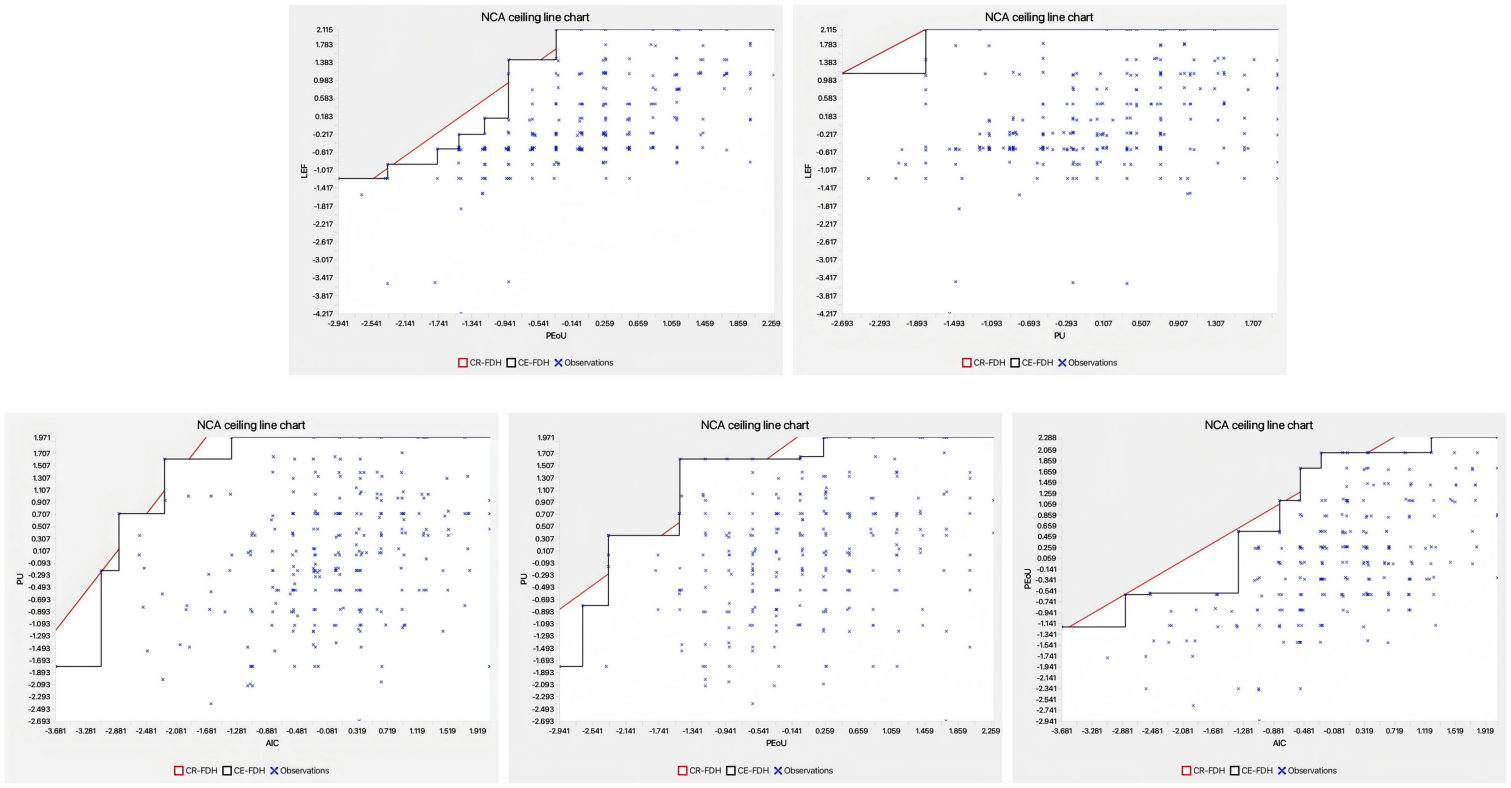
Scatter plots of necessary condition analysis.

A typical application of NCA involves two main stages. First, latent variable scores are extracted through Partial Least Squares Structural Equation Modeling (PLS-SEM), ensuring that the data fulfill the modeling requirements for multi-level statistical associations (Richter et al., 2020). Second, the NCA analysis is conducted using dedicated analytical packages integrated within statistical software such as SMART-PLS (e.g., the NCA plug-in), following standardized procedures outlined by Dul (Dul, 2016b). Specifically, NCA employs “ceiling lines” to delineate the boundaries of necessary conditions. These ceiling lines, drawn tangentially to the upper-leftmost data points within an x-y scatter plot, visually illustrate the minimal necessary level of an independent variable required to achieve certain levels of the dependent variable (see Figure 2). The strength of this approach lies in its integration of statistical rigor and visual interpretability, allowing researchers not only to rigorously test theoretical hypotheses but also to generate actionable and practical recommendations based on identified constraints.

Subsequently, we conducted a further analysis to statistically assess the significance of effect sizes (d) associated with the latent variable scores, using a bootstrapping procedure with a randomized sample size of 5,000 iterations (Dul, 2016a; Piff et al., 2015). Due to the suitability of the Ceiling Regression-Free Disposal Hull (CR-FDH) method for analyzing survey data gathered via five-point Likert scales, the results obtained from the Necessary Condition Analysis (NCA) were highly consistent with this analytical approach. The detailed results are presented in Table 7. Specifically, the NCA results revealed notable interrelationships among the constructs. For instance, the effect size of AIC on PU was found to be 0.195 (*p* < 0.001), while the effect size of PEoU on PU was 0.166 (*p* < 0.001), indicating that AIC is a necessary condition for PU. Similarly, the effect size of AIC on PEoU was 0.261 (*p* < 0.001), confirming that AIC is also a necessary condition for PEoU. Furthermore, the effect size of PEoU on LEF was 0.173 (*p* < 0.001), demonstrating that PEoU is a necessary condition for LEF.

However, the effect size of PU on LEF was only 0.015 (*p* = 0.809), failing to reach statistical significance (Table 7). Thus, PU does not satisfy the criteria for being a necessary condition for LEF. These findings collectively suggest that while AIC and PEoU are crucial for enhancing PU, and AIC is essential for improving PEoU, and PEoU is critical for enhancing LEF, PU represents an influential but not necessary factor for fostering LEF.

**Table 7:** Results of necessary condition analysis (NCA) using the CR-FDH method.

Construct	Effect size	Ceiling	Scope	Condition Inefficiency	Outcome Inefficiency	Rel. Inefficiency	Abs. Inefficiency	*p*-values
PU								
AIC	0.195	7	0.829	28.107	45.807	61.039	22.258	< 0.001
PEoU	0.166	16	0.99	45.265	39.248	66.747	16.278	< 0.001
PEoU								
AIC	0.261	8	0.815	23.747	31.561	47.813	14.398	< 0.001
LEF								
PEoU	0.173	6	1.318	43.69	38.71	65.488	21.683	< 0.001
PU	0.015	1	1.095	80.768	84.491	97.017	28.652	0.809

The bottleneck technology of LEF further clarifies the threshold levels required to achieve specific performance levels. As shown in Table 8, to reach a 40% PU level, AIC must be no less than −3.441, and PEoU must be no less than −2.906. To achieve a 40% PEoU level, AIC must be at least −3.14. To attain a 50% LEF level, AIC must be no less than −2.518, and PEoU no less than −2.347. To achieve a 100% PU level, the necessary conditions include AIC not lower than −1.683 and PEoU not lower than −0.079. Additionally, for PU to reach a 100% level, AIC must be no less than 0.71. To achieve a 100% LEF level, AIC must be no less than 0.7, PEoU no less than −0.325, and PU no less than −1.796.

**Table 8:** Bottleneck level analysis using CE-FDH method in NCA framework.

PU	AIC	PEoU	PEoU	AIC	LEF	AIC	PEoU	PU
0.00%	NN	NN	0.00%	NN	0.00%	NN	NN	NN
10.00%	NN	NN	10.00%	NN	10.00%	NN	NN	NN
20.00%	NN	NN	20.00%	NN	20.00%	NN	NN	NN
30.00%	NN	NN	30.00%	NN	30.00%	NN	NN	NN
40.00%	−3.441	−2.906	40.00%	−3.14	40.00%	NN	NN	NN
50.00%	−3.148	−2.434	50.00%	−2.498	50.00%	−2.518	−2.347	NN
60.00%	−2.855	−1.963	60.00%	−1.856	60.00%	−2.518	−1.492	NN
70.00%	−2.562	−1.492	70.00%	−1.215	70.00%	−1.124	−0.897	NN
80.00%	−2.269	−1.021	80.00%	−0.573	80.00%	−0.799	−0.897	NN
90.00%	−1.976	−0.55	90.00%	0.069	90.00%	0.07	−0.325	−1.796
100.00%	−1.683	−0.079	100.00%	0.71	100.00%	0.07	−0.325	−1.796

NN = Not Necessary, indicating that the condition is not required for the outcome to reach the specified level.

## 5 Discussion

While most hypothesized pathways in the structural model yielded statistically significant results, the relationship between Cognitive Processing Depth (CPD) and Perceived Usefulness (PU) was found to be non-significant (*β* = 0.063, *p* = 0.415). This unexpected outcome warrants careful theoretical and empirical interpretation, rather than being dismissed as merely insignificant. One possible explanation lies in the nature of shallow engagement with AI-Assisted Conversational Tools during language learning tasks. According to Lockhart and Craik (1990) Levels of Processing framework, deep cognitive engagement—such as critical evaluation, synthesis, or abstraction—has stronger effects on meaningful learning and subsequent perceived utility. However, in this study, the design of tasks and interaction with AIC may have primarily triggered surface-level processing (e.g., grammar corrections, word substitutions, or mechanical paraphrasing), which learners did not perceive as substantially beneficial in enhancing their academic performance or broader learning goals. This interpretation is also supported by recent AI-in-education research, where perceived usefulness often correlates with goal alignment and long-term transfer, rather than immediate micro-task engagement. Thus, although participants cognitively processed the content (CPD), the lack of perceived strategic value from these tools may have weakened the link to PU.

Additionally, novelty effects or tool immaturity may have contributed to this finding. Many AIC platforms are still in developmental stages and may lack domain-specific customization or feedback depth, making learners perceive them as supportive for ease of use (PEoU) but not transformative in actual academic output, thereby attenuating perceived usefulness.

In future studies, refining CPD measurement to distinguish between deep vs. shallow processing types, or supplementing quantitative results with qualitative learner feedback, may better capture how cognitive depth contributes to perceived value.

## 6 Conclusion and implication

### 6.1 Conclusion

This study employed a combined NCA and PLS-SEM approach to examine the mechanism of learning efficiency of EFL acquisition through AI-assisted conversational tools, providing a comprehensive understanding and analysis.

To ensure the robustness of the measurement model, we conducted a thorough assessment of reliability and validity. Reliability was evaluated using Cronbach’s alpha and composite reliability (CR). All constructs demonstrated high reliability, with Cronbach’s alpha and CR values exceeding the threshold of 0.7, indicating substantial internal consistency and stability of the questionnaire scales. These findings, as shown in Table 2 and Figure 2, confirmed the high reliability of the questionnaire data. The validity of the measurement model was assessed through convergent validity and discriminant validity. All indicators had average variance extracted (AVE) values exceeding 0.5, indicating robust convergent validity (Fornell and Larcker, 1981). Discriminant validity was confirmed using the Fornell-Larcker criterion and the HTMT (Heterotrait-Monotrait) ratio. The square root of each variable’s AVE exceeded its correlation coefficients with other dimensions, and the HTMT ratios were below the threshold of 0.85 (Sarstedt et al., 2014a), indicating significant discriminant validity of the measurement model.

To assess collinearity, we examined the variance inflation factors (VIF) for all predictive constructs in the structural model. All VIF values ranged from 1.692 to 2.648, well below the cutoff value of 3, indicating that collinearity was not a significant issue in the model. Subsequently, we performed bootstrapping with 5,000 subsamples to evaluate the significance of the hypotheses (Hair et al., 2021). The analysis showed that the paths from CPD to PU, from CPD × AIC to PEoU, from CPD × AIC to PU, and from COA × AIC to PEoU were not significant, thus not supporting the hypotheses. Despite these insignificant paths, the model explained a substantial amount of variance in the dependent variables: *R*^2^ for PEoU was 0.389 with a *Q*^2^ predict of 0.249; *R*^2^ for PU was 0.088 with a *Q*^2^ predict of 0.058; and *R*^2^ for LEF was 0.402 with a *Q*^2^ predict of 0.258. The *Q*^2^ values further confirmed the predictive relevance of the model, as they were all above zero. Effect size analysis (*f*^2^) indicated that multiple predictor constructs had significant effects on the dependent variables. NCA provided a unique approach to understanding complex statistical associations by identifying necessary conditions that impact the outcome variable. Unlike traditional methods, NCA quantifies the size and constraints of these necessary conditions, making it particularly adept at distinguishing “necessary but not sufficient” relationships between the dependent and independent variables (Dul, 2016b). As an enhancement to traditional sufficiency analysis, NCA offers numerical measurements of the prerequisites needed to achieve a given outcome level, thereby providing deeper insights into effect sizes and potential bottlenecks.

Initially, the PLS-SEM method was used to obtain scores for the latent variables (Richter, 2020; Richter et al., 2020). Subsequently, NCA analysis was performed using the NCA package within the Smart-PLS software, following the guidelines set by Vis and Dul (2018). The fundamental step of NCA involved plotting a ceiling line that intersected the upper-left data points on an x-y scatter plot. Figure 2 illustrates the scatter plots for all relevant relationships.

Subsequent analyses evaluated the statistical significance of the effect sizes (d) associated with the latent variable scores, using 10,000 random samples (Dul, 2016b; Piff et al., 2015). Given that the CR-FDH (Cumulative Relative Frequency - Full Disjunctive Hypothesis) line is well-suited for survey data derived from a five-point Likert scale, the interpretation of the NCA results was consistent with this method. As shown in Table 6, the results indicated that AIC (*d* = 0.195, *p* < 0.000) and PEOU (*d* = 0.166, *p* < 0.001) were necessary conditions for PU adaptation; AIC (*d* = 0.261, *p* < 0.001) was a necessary condition for PEOU; similarly, PEOU (*d* = 0.173, *p* < 0.000) was a necessary condition for LEF. However, PU (*d* = 0.015, *p* = 0.809) was not significant and thus did not constitute a necessary condition for LEF.

### 6.2 Theoretical implication

This study focuses on investigating the impact of AI-assisted conversational tools on English learning efficiency (LEF). Employing quantitative analysis centered around key variables such as perceived usefulness (PU), perceived ease of use (PEoU), interaction frequency of AI-assisted conversation (AIC), and learning efficiency (LEF), the research develops an integrated theoretical framework that synthesizes the Technology Acceptance Model (TAM) with Cognitive Load Theory (CLT). This integrative approach has yielded several important theoretical contributions. This research enriches the theoretical scope of TAM by incorporating insights derived from CLT. Traditional TAM primarily emphasizes users’ subjective acceptance intentions toward technology use. By contrast, this study further elucidates the mediating effect of cognitive processing depth (CPD)—triggered by AIC usage—on learning efficiency. This finding aligns with Gkintoni et al. (2025) cognitive load model, which posits that learning outcomes depend not merely on technological attributes but also on individuals’ cognitive processing strategies. Hence, the present research provides theoretical support for understanding the cognitive mechanisms underlying technology acceptance behaviors.

By adopting Necessary Condition Analysis (NCA), this research explicitly identifies the threshold values of key variables required to achieve specific levels of learning efficiency. For instance, to achieve a 100% level of PU, the analysis reveals that AIC must not fall below 0.71, and PEoU must not be lower than −0.079 (see Table 7). Serving as a valuable complement to Partial Least Squares Structural Equation Modeling (PLS-SEM), the NCA method effectively identifies the “bottleneck” roles of certain variables—that is, critical thresholds below which the desired outcomes become unattainable (Dul, 2016b). This methodological approach addresses the inherent limitation of traditional path analysis in failing to identify critical cut-off points, thereby enriching the boundary conditions research of TAM and CLT in practical applications. This study introduces cognitive ability (COA) as a moderating variable, revealing the role of individual differences in moderating AIC learning outcomes. Empirical results indicate that learners with higher cognitive abilities exhibit greater CPD when using AIC, thereby enhancing their learning efficiency. This finding is consistent with the theory of Self-Regulated Learning (Zimmerman, 2002), which suggests that individuals with higher cognitive capabilities are better at autonomously regulating their learning paths and strategies. The moderating role of COA underscores the necessity of considering individual differences in future technological designs and educational interventions, advocating for the creation of more cognitively adaptive AI systems. This study fills an existing research gap in AI education literature concerning non-native English learners (specifically, the EFL population). Current research on AI-assisted learning has predominantly focused on STEM disciplines and native English-speaking student populations (Luckin et al., 2016). By concentrating specifically on Chinese university students’ English learning contexts, this research expands the applicability of the TAM and CLT frameworks to cross-cultural and second-language educational environments.

The introduction of CPD and COA variables offers novel explanatory perspectives for AI educational technology research. While previous studies have frequently emphasized learning attitudes and behavioral intentions, they have largely overlooked how technological integration stimulates deeper cognitive processing pathways. By constructing an integrated model, the present study provides a new cognitive psychological interpretation of how AI technology “reshapes learning itself.” In sum, this research contributes theoretically in four significant ways: (1) deepening the understanding of AIC acceptance mechanisms through the integration of TAM and CLT; (2) employing NCA to identify critical thresholds at which variables influence learning efficiency; (3) elucidating the mediating mechanism of CPD and the moderating mechanism of COA; and (4) extending the theoretical scope of AI educational research into language learning within humanities and cross-cultural contexts. These theoretical insights not only offer a robust framework for future model optimization but also provide cognitive-level guidance for technological design and educational interventions.

### 6.3 Practical implication

With the rapid advancement of artificial intelligence technologies in educational domains, AI-assisted conversational tools have increasingly emerged as crucial supplementary resources in university-level English instruction. However, the effective implementation of such technologies is influenced not merely by their inherent sophistication, but more importantly, by their deep integration with learners’ cognitive characteristics, usage habits, and the instructional environment. This study provides several significant practical implications at multiple levels, as detailed below.

Beyond the statistical significance reported in the structural model, this study highlights a particularly noteworthy path: the positive effect of Perceived Ease of Use (PEoU) on Learning Efficiency (LEF). This result bears important pedagogical value in the context of AI-assisted conversational learning tools, especially for university-level English learners who frequently face cognitive overload and self-regulation challenges. Specifically, the finding suggests that when students perceive AIC interfaces as easy to navigate and interact with, their mental resources can be redirected from operational effort to language acquisition tasks, such as grammar refinement, argumentation, or vocabulary contextualization. This aligns with Cognitive Load Theory (CLT), which posits that reducing extraneous load—such as tool design or unintuitive input formats—can enhance germane processing and learning transfer.

Moreover, the findings indicate that cognitive processing depth (CPD) serves as a crucial mediator in the relationship between AIC use and learning efficiency. Consequently, AI tool developers should emphasize aligning system architecture with learners’ cognitive mechanisms. For instance, dialogue design could incorporate adjustable feedback modes (e.g., basic, reflective, inferential feedback), enabling the system to dynamically generate cognitively stimulating feedback based on learners’ current progress and performance, thus enhancing cognitive engagement.

Additionally, adaptive adjustment mechanisms should be incorporated into AIC systems to accommodate learners with varying cognitive abilities, as the empirical evidence confirms that cognitive ability (COA) significantly moderates learning outcomes. AI systems could automatically assess users’ cognitive capacities through historical interaction data, linguistic input quality, and task completion rates, subsequently matching appropriate interactive tasks. For example, higher cognitive-ability learners could be provided with tasks emphasizing critical thinking and language generation, whereas lower cognitive-ability students could receive more structured, scaffolded tasks to minimize cognitive load.

Furthermore, at the quantitative level, this study introduces Necessary Condition Analysis (NCA) to identify the critical threshold values for variables such as AIC, PEoU, and PU required to achieve specific levels of learning efficiency. For example, to reach a 50% LEF level, the minimum required threshold for AIC is −2.518, and for PEoU is −2.347. This analytical approach can serve as a dynamic diagnostic model for assessing instructional quality in higher education, shifting the evaluation focus from purely outcome-based indicators to process-oriented measures, thereby providing real-time insights into instructional bottlenecks. Educational managers could integrate these critical thresholds into backend monitoring systems of AI-assisted learning platforms, facilitating real-time tracking of students’ key variable performance. Timely interventions, including tailored learning recommendations and technical assistance, could thus be implemented when students persistently exhibit sub-threshold levels of AIC or PEoU, ensuring personalized instruction and differentiated service provision.

Regarding enhancing user experience and learner engagement, it is advisable to empower learners with greater control over their learning process, such as customizing their learning pace, choosing feedback frequency, and providing suggestions, thereby reinforcing the learner-centered paradigm and preventing learning apathy or technological inertia arising from excessive technological dependence. Comprehensive data ethics and privacy protection mechanisms should be established. AI systems must comply with relevant regulatory standards (e.g., the Personal Information Protection Law) when collecting and processing student data, ensuring data security, transparency of use, and controllability of outcomes. Educational institutions should also facilitate training programs and institutional arrangements to cultivate students’ healthy AI usage habits and robust data security awareness.

## 7 Limitation and future research

Although this study systematically investigates the adaptation mechanisms of AI-assisted conversational tools in university English learning contexts—constructing a comprehensive theoretical model and providing empirical evidence—it is inevitably subject to several limitations that future research should address.

First, the operationalization of AIC in this study mainly focused on interaction frequency, which may oversimplify the richness of human–AI interaction in educational settings. Prior literature has begun to emphasize that AI-assisted learning involves not just how often learners interact with AI, but also how they interact. Future research should broaden the conceptualization of AIC to include additional dimensions such as the variety of interaction types (e.g., reflective, corrective, exploratory), the functionality of AI tools (e.g., real-time adaptability, feedback provision), and the interaction quality (e.g., coherence, naturalness, user alignment). Such a multidimensional approach would enhance construct validity and provide more nuanced insights into how AI tools shape learning experiences. Second, the study employed a cross-sectional design, capturing all data at a single time point. As a result, the observed relationships among variables should be interpreted as associative rather than causal. Despite the use of Structural Equation Modeling (SEM) and Necessary Condition Analysis (NCA), which support theoretically guided directional paths, temporal or experimental confirmation is lacking. Specifically, the influence of AIC on learning efficiency may evolve nonlinearly over time. Longitudinal studies or controlled experiments are recommended for future work to assess dynamic change patterns and infer causality more robustly.

Although our sample of 297 university students is statistically adequate and demographically diverse, its composition is limited to a specific educational and regional context. Cultural, technological, and pedagogical differences may limit the generalizability of our findings. Future research should recruit participants from various institutional types, regions, and even international contexts to test the cultural robustness of the model. While the study focused on five key constructs—perceived usefulness (PU), perceived ease of use (PEoU), frequency of AI-assisted conversation (AIC), cognitive processing depth (CPD), and learning efficiency (LEF)—several potentially important mediators and moderators were not included. For example, learning motivation, task type, peer interaction, and instructor feedback may significantly influence the effects of AIC on learning outcomes. Additionally, while CPD was hypothesized as a cognitive mediator, its non-significant effect on PU may be explained by shallow cognitive processing in repetitive task settings—a direction worth further investigation. Future studies should incorporate richer motivational, contextual, and cognitive-affective factors to deepen the explanatory power of the model.

Finally, the presentation of results should not solely emphasize statistical significance but also clearly explain their pedagogical implications. For instance, the significant effect of PEoU on LEF indicates that AI tools should be designed to reduce cognitive load, particularly for students with limited language proficiency or digital literacy. Such practical insights should be integrated into future AI educational tool development. Some formatting and language issues (e.g., inconsistent capitalization in subheadings, acronym overuse) were noted and have been revised. Future research should continue to improve clarity, reduce technical jargon, and provide consistent definitions across text, tables, and figures to ensure transparency and readability.

## Data Availability

The original contributions presented in the study are included in the article/supplementary material, further inquiries can be directed to the corresponding author/s.
